# Photodynamic Therapy in Pediatric Dentistry

**DOI:** 10.1155/2014/217172

**Published:** 2014-10-02

**Authors:** Patricia da Silva Barbosa, Danilo Antônio Duarte, Mariana Ferreira Leite, Giselle Rodrigues de Sant' Anna

**Affiliations:** ^1^University Cruzeiro do Sul (UNICSUL), Avenida Frederick Hoffamam, 188 Jardim Coimbra, 03693040 São Paulo, SP, Brazil; ^2^University Cruzeiro do Sul (UNICSUL), Rua Galvão Bueno, 868 Liberdade, 1506-000 São Paulo, SP, Brazil; ^3^Dentistry School, University Cruzeiro do Sul (UNICSUL), Avenida Doutor Ussiel Cirilo 225, 08060-070 São Miguel, SP, Brazil; ^4^Dentistry School, University Cruzeiro do Sul (UNICSUL), Rua Saturnino dos Santos, 106 Vila Firminiano Pinto, 04124-150 São Paulo, SP, Brazil

## Abstract

Conservation of deciduous teeth with pulp alterations caused by caries and trauma is a major therapeutic challenge in pediatric dentistry as a result of the internal anatomy and life cycle characteristic. It is essential that the root canal procedures sanitizers have a performance in eliminating bacterial. In this context, antimicrobial photodynamic therapy (PAT) is promising and emerging as adjuvant therapy in an attempt to eliminate the microorganisms persistent to chemi-mechanical preparation. Since there is presence of oxygen in cells, photosensitizer activated by light can react with molecules in its vicinity by electrons' or hydrogen's transfer, leading to microorganism death. This paper reports the case of 4-year-old patient, female, with early childhood caries. The proposed endodontic treatment incuded chemomechanical treatment allied to PAT in the decontamination of root canals using methylene blue dye 50 *μ*g/mL during 3–5 minutes and 40 J/cm^2^ as energy density, taking into account the need for tissue penetration and effectiveness of PAT inside the dentinal tubules.

## 1. Introduction

Photodynamic therapy (PDT) involves the use of a photosensitizer that is activated by exposure to light of a specific wavelength in the presence of oxygen. The transfer of energy from the activated photosensitizer to available oxygen results in toxic oxygen species formation, such as singlet oxygen and free radicals. These very reactive chemical species can damage proteins, lipids, nucleic acids, and other cellular components. Applications of PDT in dentistry are growing rapidly: treatment of oral cancer and bacterial and fungal infection therapies.

Advances in tooth decay prevention have been translated into a reduction in its incidence and prevalence, and despite the efforts in this direction and understanding of the importance of maintaining the deciduous dentition healthy, often there are a high number of deep carious lesions in children where the disease is polarized, with pulp involvement. About 75% of primary teeth affected by carious process, with medium and high activity, reveal a pulp involvement [1]. This is due to thinner enamel and dentin present in primary teeth and lower enamel mineralization compared to permanent teeth, as well as the presence of prominent pulp horns, located under the cusps and less vestibular-lingual distance between them and finally an extreme neck constriction present in these teeth [2].

Indeed, traumatic injuries, especially in anterior teeth, have a high prevalence in pediatric dentistry [3], thus becoming a serious problem, because the pulpal involvement that usually take is an issue, as well as, the patient emotional condition for himself and for their carers.

The maintenance of primary teeth with pulp changes caused by caries or trauma is a major therapeutic challenge in pediatric dentistry because of the pulpal biological cycle characteristic of these teeth as well as internal anatomy, hence the need for sanitizers root canal procedures that have a high performance in eliminating bacterial, since this leads to the success. Most failures or unsuccessful endodontic treatment is related to the persistence of microorganisms that survived the chemomechanical preparation or medications and dressings [4].

**Figure 1:**
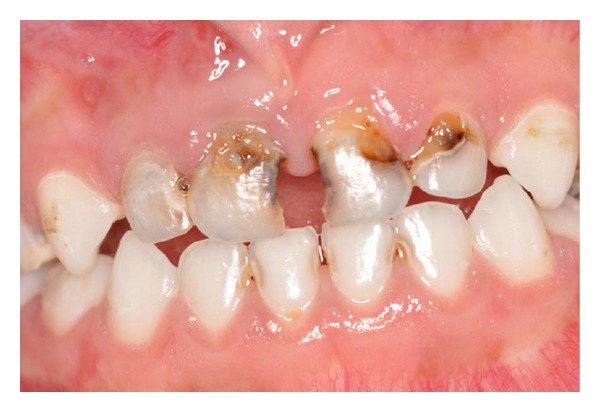
Clinical aspect of early childhood caries.

**Figure 2:**
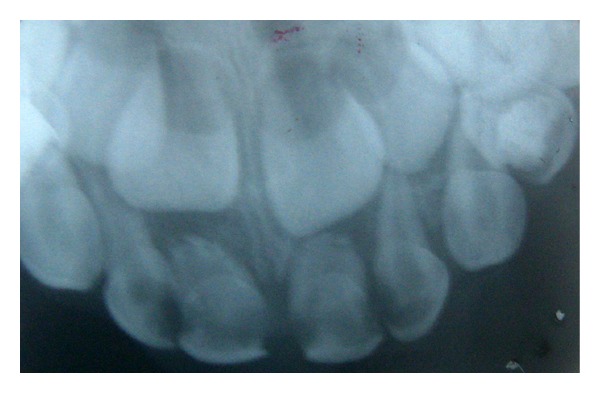
Radiographic image presenting periapical lesion and increased pericementary space.

The pathological pulp processes are commonly found in deciduous teeth (Figures 1 and 2). In these processes anaerobic microorganisms were quantified in 96.7% of cases, black-pigmented bacilli in 35.5%, aerobic in 93.5%,* streptococci *in 96.7% and* S. mutans *in 48.4%, constituting a polymicrobial ethiology infection [5].

In this context antimicrobial photodynamic therapy (PAT) is a very promising approach to disinfect dentine [6, 7] since in the presence of oxygen found in cells, the photosensitizer activated by light can react with molecules by electrons or hydrogen transfer, leading to free radicals production (type I reaction) or by energy transfer to oxygen (type II reaction), leading to singlet oxygen production. Both paths can lead to cell death, in this case, microbial [8–10]. One PAT advantage is that the resistance to it by microorganisms seems unlikely, since in microbial cells, singlet oxygen, and free radicals interact with various cellular structures and metabolic pathways. The PAT is also effective against bacteria resistant to antibiotics and antibiotic susceptible, and repeated photosensitization has no led to selection of resistant strains [11]. In 2014 de Sant'Anna reported that PAT provides favorable prognosis when used as an adjunct to conventional treatment related to diabetic pediatric patients [12].

Thus, PAT has emerged as adjuvant therapy for endodontic treatment in an attempt to eliminate the microorganisms persistent chemical-mechanical preparation. Several studies [4, 12–24] have investigated PAT activity in pulp diseases related with bacteria [4, 13–24]. It is observed in these studies [4, 12–24] 70% reductions of viable bacteria, with better success by partnering conventional treatment and PAT.

It should be noted when PAT is an option that some principles should be followed, among them preirradiation time between 3–5 minutes to sensitize the biofilm bacteria involved. Another point is energy density that takes into account the characteristic of the tissue and the penetration needed to effectiveness of PAT within the dentinal tubule.

In the establishment of protocols employed in PAT in endodontics, we highlight the association of laser in the red spectrum with blue photosensitizers, since this type of light acts in bone repair in the presence of periapical pathology in permanent teeth and in the furcation area in primary teeth, increasing bone repair associated with radicular dentin decontamination [25, 26].

This clinical case report will present step by step the use of PAT as an adjuvant in endodontic treatment of deciduous tooth.

## 2. Case Report and Discussion

**Figure 3:**
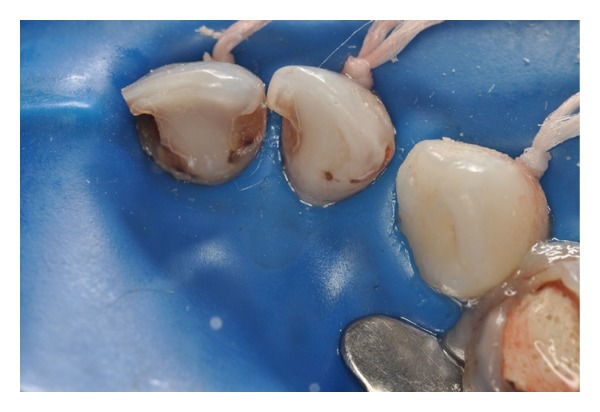
Early childhood caries (lingual aspect).

Patient MQF, female, 4-year-old attended the service of Pediatric Dentistry Health Department of Barueri City because of poor oral health. In clinical and radiographic examination, carious lesions were observed in the elements 55, 54, 53, 52, 51, 61, 62, 63, 64, 74, 75, 72, 71, 81, 82, 84, and 85 (Figures 1 and 2). Endodontic treatment is indicated for the elements 54, 52, 51, 61, 62, 64, and 74 (Figure 3).

On radiographic examination there was an increase of pericementary space and radiolucent lesions in periapex (Figure 2).

**Figure 4:**
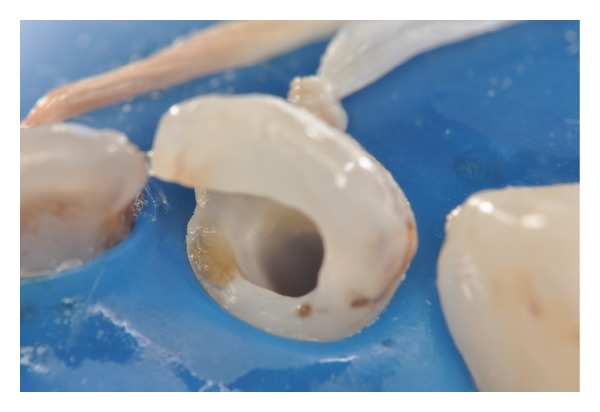
Root canal opened with spherical bur in high and low speed.

**Figure 5:**
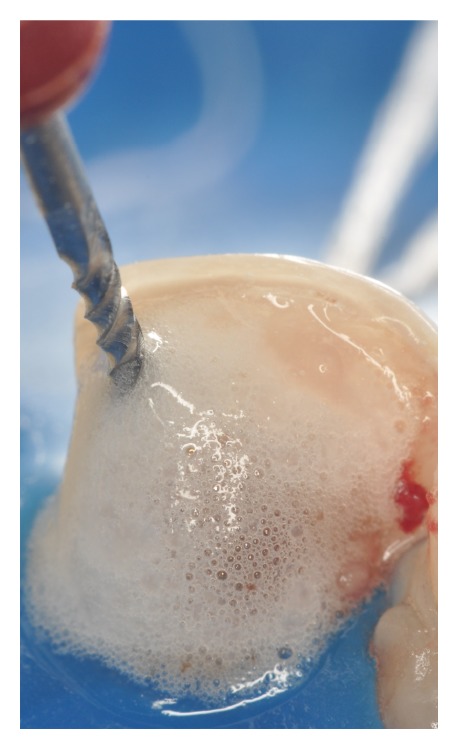
Chemical-mechanical preparation using K-flex file with endo-PTC (ASFER, Chemical Ind, São Caetano do Sul, SP, Brazil) and NaClO 1%.

**Figure 6:**
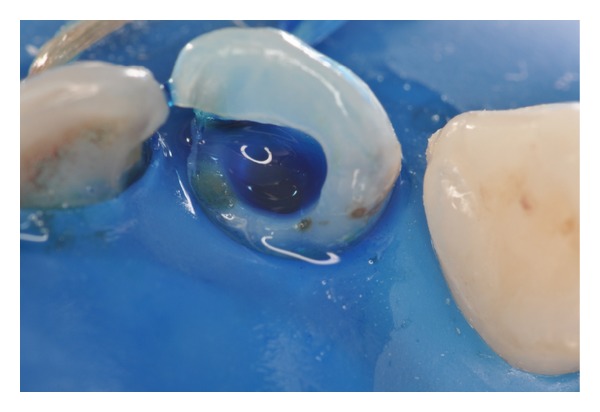
Use of methylene blue solution (50 *μ*g/mL) as a photosensitizing agent and preirradiation time of 5 minutes.

**Figure 7:**
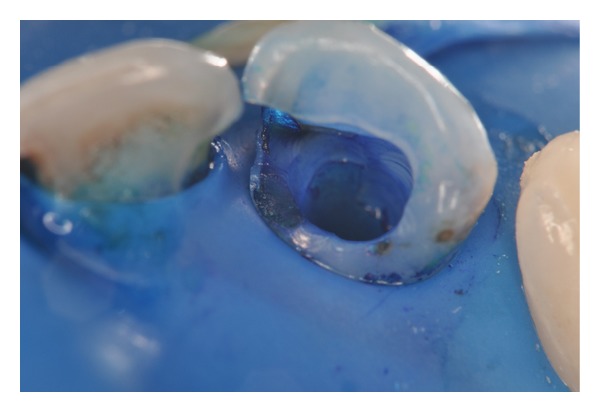
Dentin tissue stained with photosensitizing agent. It is important to aspirate the contents prior to light irradiation.

**Figure 8:**
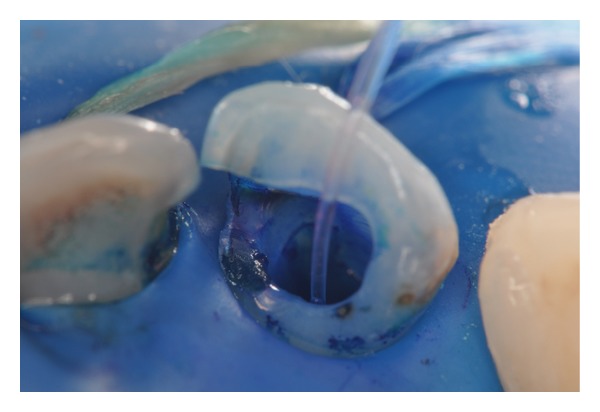
Optical fiber positioned and irradiation of red laser (40 J/cm^2^).

**Figure 9:**
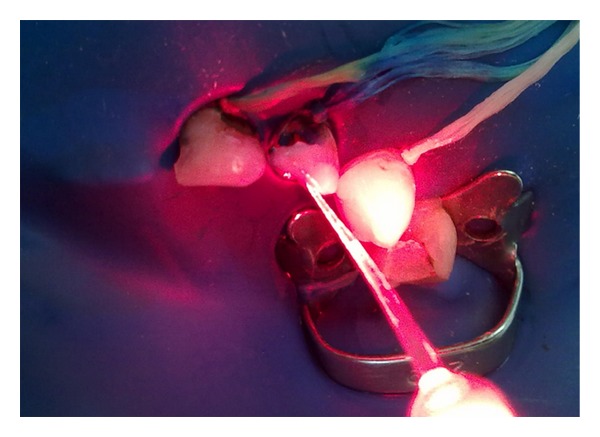
Laser irradiation.

A combined endodontic therapy using conventional methods for root canals sanitization through mechanical and chemical preparation (Figures 4 and 5) and PAT (Figures 6, 7, 8, and 9) associated was proposed, in an attempt to eliminate as many bacteria as possible from the root canal, starting the process of oral environment adequation with endodontic treatment of elements 61 and 62. The conventional endodontic treatment was performed using endodontic files (first +3) k type flexofile 21 mm (Dentisply Maillefer, York, PA, USA). The manual instrumentation was performed with endo PTC (tween 80, carbowax and urea peroxide) and 1% NaOCL. Immediately after convencional treatment, PAT was performed using methylene blue 50 *μ*g/mL as photosensitizer for 5 minutes as pre irradiation time and after this red laser was delivered using an optical fiber with 40 J/cm^2^ of fluence [27].

The therapeutic goal of each root treatment is creation of a sterile, bacteria-free environment both in the tooth, at the apex, including the periodontal tissue and the surrounding apical bone. Only then osteoblasts would be able to complete the healing process in the apical area in primary teeth. There are two factors that complicate achieving sterility in the tooth: the anatomical root configuration and the special characteristics of the resident bacterial flora. It is the presence of bacteria in the dentinal tubules that is considered to be one of the main causes of root canal failures.

**Figure 10:**
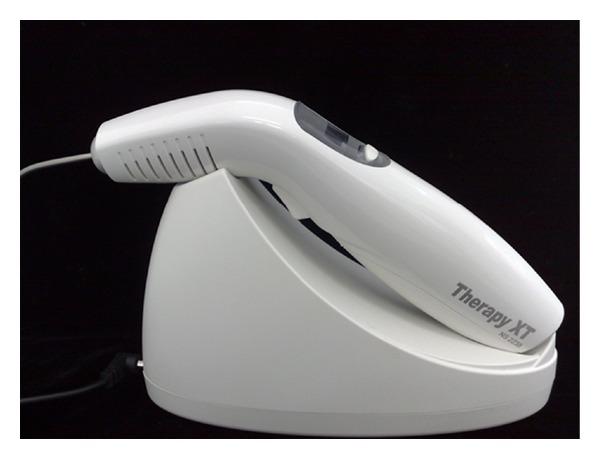
Laser unit used (*λ* = 660 nm) and optical fiber (Therapy XT-DMC, São Carlos, SP, Brazil).

Many types of lasers have been used for this particular purpose, but only the wavelengths which can deliver their power through extremely fine flexible fiber optic systems (Figure 10) and penetrate dentin to a depth that can eliminate bacteria are applicable. Laser light with wavelength in the near infrared range is absorbed by dentin only to a small extent. This characteristic is used for root canal sterilization as we do not want superficial absorption in the dentin, but a deep penetration into the intertubular and intratubular tissue, in order to produce a sufficient bactericidal effect in the deep layers [25]. Photodynamic therapy is included in this context as a potentially bactericidal approach adjunct to conventional treatment.

As noted previously, several studies have investigated the performance [4, 13–23] with significant reductions in viable bacteria and better success by partnering conventional treatment and PAT. The photosensitizing agent can be presented in the pharmaceutical form of a solution as used in this case or aqueous gel. It should be emphasized that, in both forms, the application can be processed easily; however, in solution form the tissue is more intensified impregnated with the dye and is easier its removal prior to laser irradiation.

**Figure 11:**
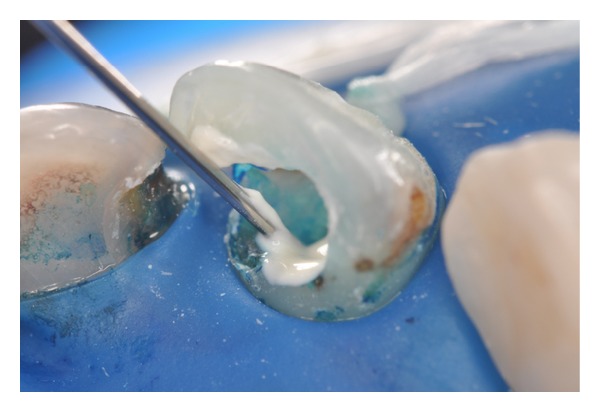
Root canal filling using calcium hydroxide Calen (SSWhite Duflex, Rio de Janeiro, RJ, Brazil).

**Figure 12:**
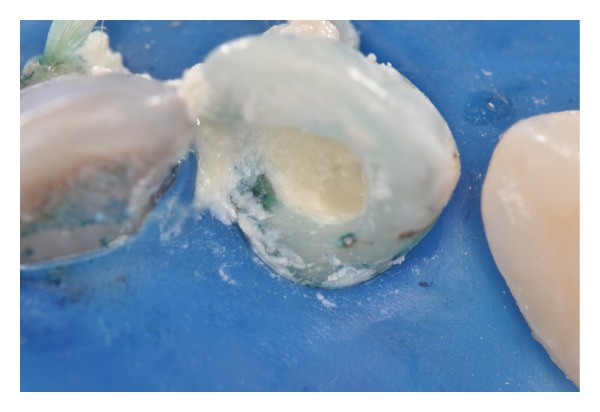
Root canal filling.

**Figure 13:**
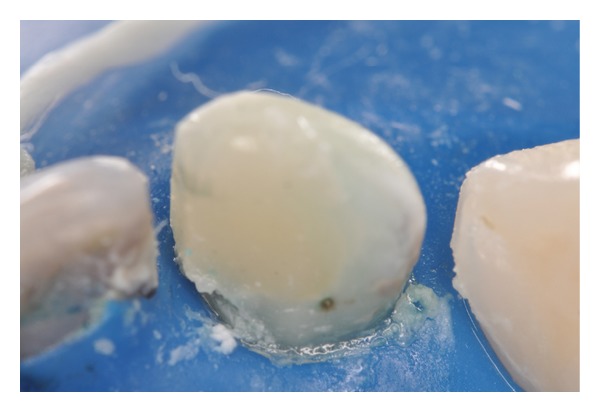
Temporary restoration with high viscosity glass ionomer cement Maxxion R (FGM Produtos Odontológicos, Joinville, SC, Brazil).

**Figure 14:**
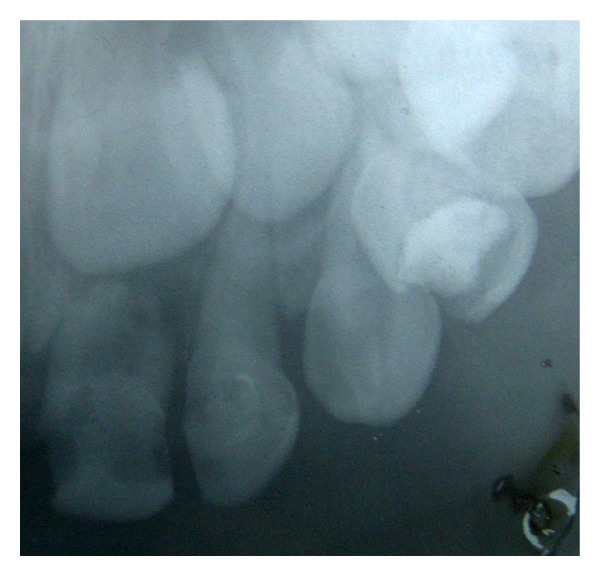
Final radiographic image.

In this case report our option as filling material was calcium hydroxide (Figures 11 and 12) and then the tooth was restored with glass ionomer cement (Figures 13 and 14).

In pediatric dentistry is worth noting that much of the procedures success is tied to the behavioral management; this in turn is also linked to operative time and hence the need to use a light source (Figure 10) that allows short exposure times, once endodontic therapy per se corresponds to a lengthy procedure.

Modern laser technology and therapies associated have brought crucial advantages to successful techniques, beyond those of conventional endodontics. Based on evidences [4, 13–22] PAT can provide favorable prognosis with substantial bacterial reduction with an interesting time-cost and benefits relations for dentistry not different for pediatric dentistry.
